# Congenital Extrahepatic Portosystemic Shunt Complicated by the Development of Hepatoblastoma: A Case Report and Review of Literature

**DOI:** 10.7759/cureus.54508

**Published:** 2024-02-20

**Authors:** Mira AlMheiri, Hala B Mrayyan, Balaji Krishnamurthy, Ajay P Dsouza

**Affiliations:** 1 Pediatric Gastroenterology, Al Jalila Children's Specialty Hospital, Dubai, ARE; 2 Gastroenterology and Hepatology, Dubai Medical College, Dubai, ARE; 3 Radiology, Al Jalila Children's Specialty Hospital, Dubai, ARE

**Keywords:** rare disease, gastroenterology and hepatology, hepatoblastoma, congenital portosystemic shunts (cpss), congenital extrahepatic portosystemic shunts (ceps), abernethy malformation

## Abstract

Congenital portosystemic shunts (CPSS) or congenital extrahepatic portosystemic shunts (CEPS) is a rare malformation. This congenital anomaly presents with a diverse array of clinical manifestations, ranging from asymptomatic to severe complications such as cardiac failure, pronounced pulmonary hypertension, and widespread pulmonary arteriovenous malformations. CPSS increases the risk of developing benign or malignant liver tumors, including nodular regenerative hyperplasia, focal nodular hyperplasia, hepatic adenoma, hepatocellular carcinoma, and hepatoblastoma.

We report a case of a 15-month-old boy, identified with Abernethy's malformation type Ib, who presented with an abdominal mass during a follow-up. A comprehensive assessment established a diagnosis of hepatoblastoma. The patient was transferred to a specialized liver transplant center for further treatment and management. This is a review of literature highlighting the complexity of Abernethy malformation and its associated risk of liver tumors.

## Introduction

The congenital portosystemic shunt (CPSS) was first described by John Abernethy in 1793. The portal system transfers the blood from intraperitoneal gastrointestinal organs to the liver.

Abernethy malformation causes bypassing of intestinal and splenic blood around the liver with direct drainage into the systemic system. There are two types of shunts: type I is defined by the complete diversion of portal blood into the vena cava with an associated congenital absence of the portal vein; and type II is characterized by an intact but diverted portal vein through a side-to-side extrahepatic connection to the vena cava [1,2]. Consequently, toxins from the intestine bypass the liver causing hepatic encephalopathy, pulmonary hypertension, hepatopulmonary syndrome, and increased risk of hepatic malignancies [3].

Identifying the presence or absence of hepatic portal venous supply holds significance, as it can influence the available treatment options. Different therapeutic approaches can be used such as embolization, transcatheter closure of the shunt, or liver transplant.

## Case presentation

The child was born prematurely at 32 weeks of gestation. He was shifted to the Neonatal Intensive Care Unit (NICU) after birth in view of respiratory distress and prematurity. The X-ray was suggestive of a tracheoesophageal fistula (TEF) that was repaired surgically in his early neonatal life. His echocardiography (echo) showed a large patent ductus arteriosus (PDA), with a left-to-right shunt, and an ostium secundum atrial septal defect (ASD). The PDA was treated medically with paracetamol.

**Figure 1:**
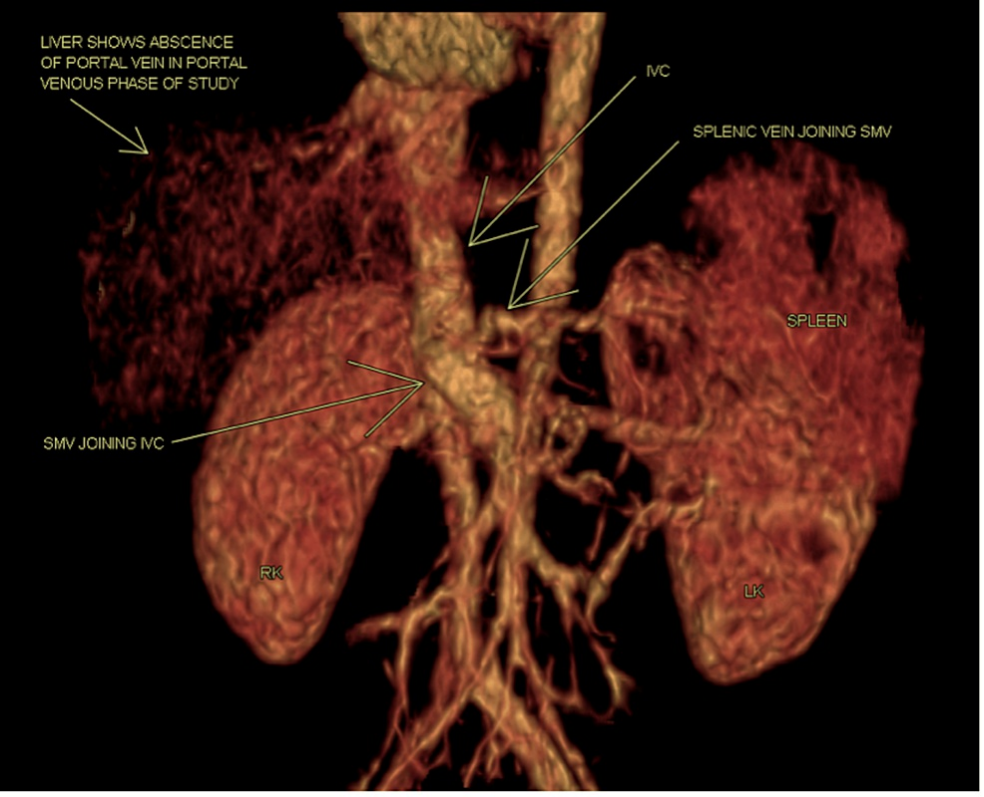
3D reconstruction CT of the portosystemic shunt (anterior view)- the superior mesenteric vein and the splenic vein are draining into the IVC with an absent portal vein. 3D: three-dimensional; CT: computed tomography; IVC: inferior vena cava; SMV: superior mesenteric vein

During a routine abdominal ultrasound, an incidental finding of hepatic portosystemic shunt was identified, and computed tomography (CT) revealed an abnormal connection of the mesenteric and splenic veins to the inferior vena cava (IVC), with both veins draining into the IVC, resulting in a portosystemic shunt with no visualization of the portal vein at the porta hepatis, nor intrahepatic branches of the main portal vein; the aforementioned findings were supported by a 3D (three-dimensional) reconstruction CT (Figure 1).

Upon follow-up, he was well. However, the parents noticed fullness of the upper abdomen. Physical examination revealed a palpable firm mass on the right side of the abdomen but no splenomegaly.

His blood test showed slightly elevated transaminases: aspartate-aminotransferase (AST) at 129 U/L and alanine-aminotransferase (ALT) at 53 U/L. The hepatic synthetic function was preserved. Notably, alpha-fetoprotein (AFP) was significantly elevated, exceeding 20,000 ng/mL.

**Figure 2:**
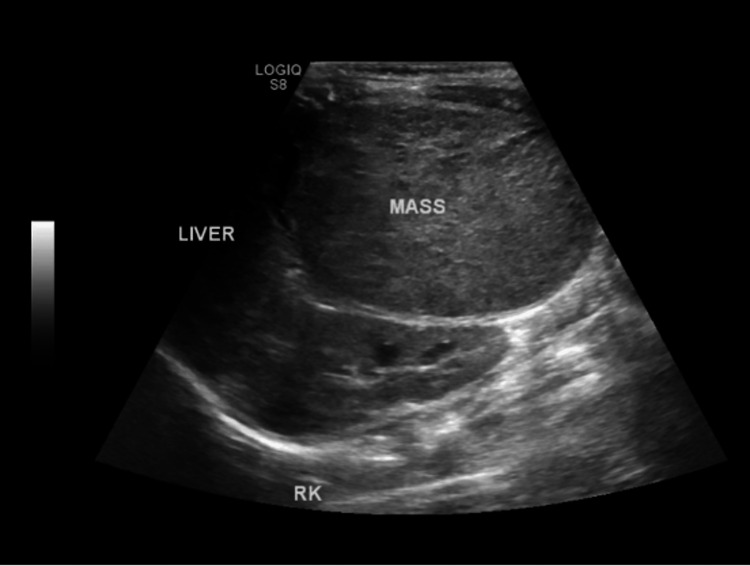
Ultrasound of the abdomen (axial view)- an isoechoic liver mass, measured 9.80 × 9.32 × 6.78 cm.

**Figure 3:**
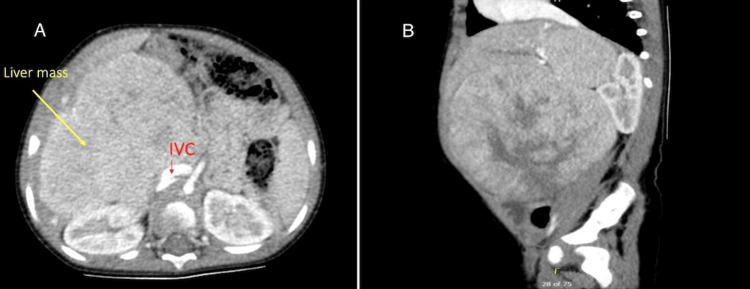
CT scan of the abdomen (axial and sagittal views)- a heterogeneous mass in the right lobe of the liver, compressing on the IVC. A: axial view; B: sagittal view

**Figure 4:**
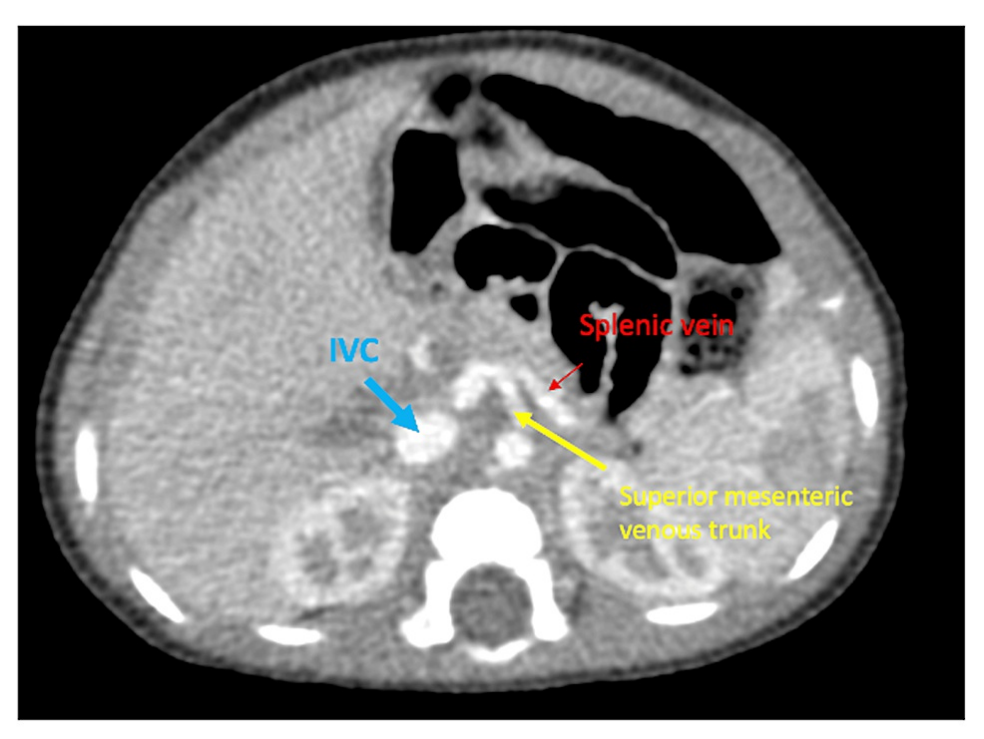
CT scan of the abdomen (axial view): confluence of the superior mesenteric venous trunk and the splenic vein into the IVC. CT: computed tomography; IVC: inferior vena cava

The abdominal ultrasound identified an isoechoic lesion located at the right upper quadrant, inevitably originating from the liver with an approximate measurement of 9.8 cm × 9.32 cm × 6.78 cm (Figure 2). Color flow Doppler demonstrated heightened vascularity within the lesion, surrounded by a thin, well-defined capsule. The adjacent liver exhibited normal echogenicity, with no evidence of ascites. A subsequent CT scan of the chest and abdomen revealed a substantial mass located in the right lobe of the liver, characterized by an enormous extrahepatic component (Figures 3A-3B). The intrahepatic portion was positioned between the right and middle hepatic veins, exerting pressure on the IVC. Throughout the portal venous phase, the mass displayed a heterogeneous enhancement, with non-enhancing areas suggestive of necrosis. Notably, the major portal vein was not visualized. The mesenteric venous tributaries anastomosed to generate a large superior mesenteric venous trunk, which merged with the splenic vein. This trunk was then connected to the IVC via an ultrashort segment (Figure 4). The biopsy of the mass confirmed hepatoblastoma, epithelial type and with both embryonal and fetal subtype, tumor staging was PRETEXT (PRE-Treatment EXTent of tumor) stage II.

The case was discussed in a multidisciplinary meeting with pediatric surgery, oncology, and hepatology teams, as well as in an international oncology tumor board meeting. Before starting the treatment plan for our patient, tumor risk stratification was applied based on the location, metastasis, and AFP level. Our case was classified into standard risk, as he had an underlying Abernethy’s malformation, we proceeded with SIOPEL III protocol with preoperative chemotherapy of cisplatin, then progressed with a liver transplant. The patient completed chemotherapy courses and successfully had a liver transplant, without complications.

## Discussion

Abernethy's malformation or CPSS is a rare congenital vascular anomaly in the splanchnic venous system where the blood from the intestine bypasses the liver directly to the vena cava, azygos vein, or right atrium [4]. The portal venous system develops through the fourth to tenth weeks of gestation. Portosystemic shunt is classified into two types of shunts: type I is defined by the complete diversion of portal blood away from the liver toward the systemic veins (such as the renal veins, iliac veins, or IVC) through an end-to-side shunt, with an associated congenital absence of the portal vein. It is subdivided into type Ia where the splenic and superior mesenteric veins flow into distinct systemic veins; while type Ib has a short extrahepatic portal vein, formed by the union of the splenic and superior mesenteric veins, which empties into a systemic vein. Type II shunt is a partial shunt, where the blood flows to the liver through a hypoplastic portal vein before emptying into the IVC through an extrahepatic side-to-side shunt [2,5]. In this case, a CT scan showed type Ib Abernethy malformation.

Reported cases exhibited an association with other congenital malformations. In a systemic review of 61 reported cases, 19 patients had congenital heart disease (CHD), seven had biliary atresia, and five had skeletal anomalies [6]. In the 2021 cohort of 11 patients with CPSS, five patients had congenital cardiac diseases, four had Turner syndrome, and four had intestinal malrotation.

Additional case series described two patients with CPSS along with associated congenital anomalies, a three years old with a tethered spinal cord, microcephaly, and interatrial septal aneurysm (ASA), and a two-year-old with TEF and small ventricular septal defect (VSD), which is similar to our case which he had CHD and TEF [7].

CPSS can be diagnosed at any age, in a systemic review of 22 patients, age at diagnosis ranged from prenatal to 84 years; 66% were diagnosed before 12 years of age and 24% in adulthood. Clinical presentation is variable, can be asymptomatic incidentally found during imaging, or can present during the neonatal period with cholestasis and abnormal newborn screening for galactosemia. Shunting can be associated with hyperammonemia and hepatic encephalopathy, causing neurological symptoms ranging from learning difficulties to attention-deficit disorders (ADD) and seizures [8].

In the literature, we observed that there is an association between CPSS and liver lesions, initial reported cases upon further examination were identified with benign in-nature lesions including focal nodular hyperplasia (FNH) and nodular regenerative hyperplasia (NRH). However, few cases developed malignant liver tumors such as hepatoblastoma and hepatocellular carcinoma, more commonly seen in type I Abernethy malformation. Although there is no clear understanding of the pathogenesis, it is attributed to the imbalance in portal venous flow to the liver resulting in reactive hepatic hypertrophy [9]. In a case series of 22 children with Abernethy malformation, six developed FNH or NRH, and two developed hepatoblastoma [7]. In another systematic review, from 316 reported cases, 82 developed liver masses, 12 of which were malignant with four patients with hepatoblastoma and eight patients with hepatocellular carcinoma, of which three of the eight cases were under 12 years of age. One hundred forty-two patients required either interventional or surgical treatment, 32 of them received liver transplants, and 110 underwent shunt closure or reduction procedures [8].

CPSS treatment is challenging, in some cases spontaneous closure of the shunt has been reported. Others required immediate intervention given the severity of the symptoms. In most patients with type I shunt, with associated hyperammonemia, hepatopulmonary syndrome, or liver lesions, a liver transplant should be considered [10].

In hepatoblastoma with PRETEXT II stage, treatment with either primary resection or neoadjuvant chemotherapy followed by resection is the preferred option. However, as our case had an underlying Abernethy’s malformation, we decided to start chemotherapy SIOPEL III protocol, then proceed with liver transplantation rather than liver resection of the tumor because of the high risk of liver failure following liver resection, due to poor portal circulation. Our patient was transferred to the liver transplant center for liver transplantation.

There are no current guidelines regarding surveillance and monitoring of liver tumors in patients with CPSS, most centers recommend monitoring with serial liver function tests, AFP, and imaging of the liver. A consensus guideline needs to be established to determine the best approach for managing these rare cases.

## Conclusions

In summary, Abernethy's malformation, characterized by CPSS, poses a rare and complex medical challenge with a spectrum of clinical presentations, especially when presenting with malignancy. This case report underscores the critical importance of early identification, accurate diagnosis, and personalized treatment strategies. There is a need for heightened awareness and multidisciplinary collaborations on Abernethy's malformation.
